# Novel Mannich bases with strong carbonic anhydrases and acetylcholinesterase inhibition effects: 3-(aminomethyl)-6-{3-[4-(trifluoromethyl)phenyl]acryloyl}-2(3H)-benzoxazolones

**DOI:** 10.3906/kim-2101-25

**Published:** 2021-06-30

**Authors:** Sinan BiLGiNER, Barış ANIL, Mehmet KOCA, Yeliz DEMİR, İlhami GÜLÇİN

**Affiliations:** 1 Department of Pharmaceutical Chemistry, Faculty of Pharmacy, Atatürk University, Erzurum Turkey; 2 Department of Chemistry, Faculty of Science, Atatürk University, Erzurum Turkey; 3 Nihat Delibalta Göle Vocational High School, Ardahan University, Ardahan Turkey

**Keywords:** Acetylcholinesterase, carbonic anhydrase, chalcone, Mannich bases, molecular docking

## Abstract

In this study, a new series of Mannich bases, 3-(aminomethyl)-6-{3-[4-(trifluoromethyl)phenyl]acryloyl}-2( *3H* )-benzoxazolones ( **1a–g** ), were synthesized by the Mannich reaction. Inhibitory effects of the newly synthesized compounds towards carbonic anhydrases (CAs) and acetylcholinesterase (AChE) enzymes were evaluated to find out new potential drug candidate compounds. According to the inhibitory activity results, K_i_ values of the compounds **1** and **1a–g** were in the range of 12.3 ± 1.2 to 154.0 ± 9.3 nM against hCA I, and they were in the range of 8.6 ± 1.9 to 41.0 ± 5.5 nM against hCA II. Ki values of acetazolamide (AZA) that was used as a reference compound were 84.4 ± 8.4 nM towards hCA I and 59.2 ± 4.8 nM towards hCA II. K_i_ values of the compounds **1** and **1a–g** were in the range of 35.2 ± 2.0 to 158.9 ± 33.5 nM towards AChE. K_i_ value of Tacrine (TAC), the reference compound, was 68.6 ± 3.8 nM towards AChE. Furthermore, docking studies were done with the most potent compounds **1d** , **1g** , and **1f** (in terms of hCA I, hCA II, and AChE inhibition effects, respectively) to determine the binding profiles of the series with these enzymes. Additionally, the prediction of ADME profiles of the compounds pointed out that the newly synthesized compounds had desirable physicochemical properties as lead compounds for further studies.

## 1. Introduction

Alzheimer’s disease (AD) is a devastating, multifactorial, chronic, and progressive neurodegenerative disease that causes dementia [1]. The fact that it is affecting 46.8 million people worldwide today indicates that AD is one of the most important health problems to be treated [2]. Although the disease cannot be cured, its progression can be stopped. According to the cholinergic hypothesis, which is one of the theses to understand the pathogenesis of the disease, losses occur both in the level of acetylcholine and cholinergic neurons in the cerebral cortex in AD [3–5]. For this purpose, many cholinesterase inhibitors such as donepezil, tacrine, and rivastigmine are used in the clinic. However, these drugs in the market have many side effects like nausea, diarrhea, hepatotoxicity, and vomiting [6]. Thus, there is a need for novel compounds having AChE inhibition effects with no or reduced side effects for the treatment of AD.

Carbonic anhydrases (CAs) are widespread zinc enzymes that are related to many important physiological and pathological processes via the hydration of carbon dioxide to bicarbonate [7,8]. To date, genetically eight different CA families have been determined as α-, β-, γ-, δ-, ζ- η-, θ-, and ι-CAs [9,10]. α-CA, which has 16 different isozymes, is involved in many different tissues and organs in mammals [11,12]. Thus, α-CA isoenzymes are used as drug targets in medicinal chemistry for the treatment of many diseases [12]. There are so many studies in the literature that report human carbonic anhydrase (hCA) inhibitors or activators with potential use as diuretic, anticancer, antiglaucoma, antiobesity, and antiinfective compounds [6,13–15]. Among hCAs, hCA I has an important function in the regulation of retinal edema [15,16]. Besides, hCA II isozyme inhibitor compounds are used in clinics as antiedema, antiglaucoma, and antiepileptic agents [4,12,15–18]. However, the hCA inhibitors used have undesired side effects because of having low selectivity to these isoenzymes [12,16–18]. As a result, there is a need in clinics for novel CA inhibitory compounds with higher inhibitory activity and selectivity to any of hCAs. 

**Figure 1:**
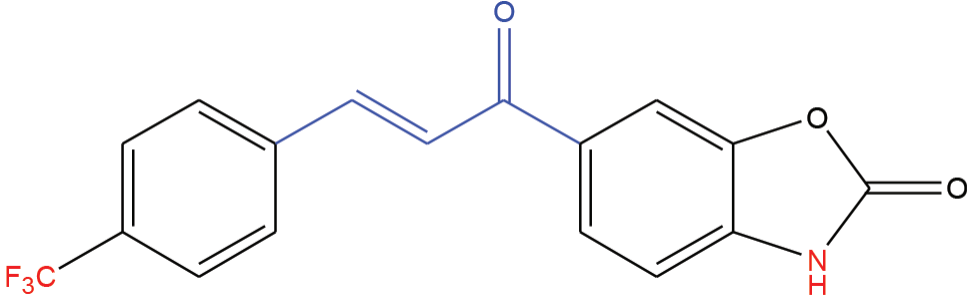
Structure of the previously synthesized compound showing high inhibitory activity towards both hCA I and hCA II enzymes.

The design of drugs bearing chalcones is often used in medicinal chemistry due to their several bioactivities such as antiinflammatory [19], antifungal [20,21], antimalarial [22,23], antimicrobial [24], anticancer [25,26], cytotoxic [10,27], antioxidant [28,29], carbonic anhydrase inhibiting [10,27,30], acetylcholinesterase inhibiting [31–34], and antidiabetic [35,36] activities. In our previous studies, we synthesized a series of benzoxazolone containing chalcone compounds and evaluated their cytotoxic and CA-inhibiting activities [27]. Among the series, trifluoromethyl derivative compound, 6-[3-(4-trifluoromethylphenyl)-2-propenoyl]-2( *3H* )-benzoxazolone (Figure 1), showed high inhibitory activities towards both hCA I and hCA II enzymes. For further studies, the previously synthesized chalcone compound presented in Figure 1 was planned to be derivatized from the 3rd position of the benzoxazolone ring by Mannich reaction. 

Mannich bases are known as an important class in medicinal chemistry with various biological activities such as cytotoxic [25,37], antibacterial [38,39], antifungal [21,40], carbonic anhydrase inhibitory [41–44], and acetylcholinesterase inhibitory [42,45,46] activities. Moreover, Mannich bases are frequently used in drug design in medicinal chemistry because they alter the pharmacokinetic properties of compounds. The hydrophilic properties of compounds can be increased by adding a polar function to the structures of the compounds via aminomethylation (Mannich reaction) [47–49].

As conclusion, we first aimed to synthesize novel Mannich bases of the previously synthesized chalcone compound, 6-[3-(4-trifluoromethylphenyl)-2-propenoyl]-2( *3H* )-benzoxazolone, via aminomethylation from the 3rd position of the benzoxazolone ring. Carbonic anhydrase (CA) and acetylcholinesterase (AChE) inhibitory activities of the compounds were evaluated to find out new potential drug candidate molecule/s. Furthermore, docking studies were done to confirm and explain the inhibition effects of the newly synthesized compounds.

## 2. Materials and method

### 2.1. Chemistry

All chemicals and solvents were Sigma-Aldrich (Germany) and Merck (Germany). Bruker AVANCE III 400 MHz (Bruker, Karlsruhe, Germany) spectrometer [400 Hz (^1^H) and 100 Hz (^13^C)] was used to record the nuclear magnetic resonance (NMR) spectra (^1^H NMR, ^13^C NMR). Dimethyl sulfoxide (DMSO)-d6 was used as a solvent. NMR VTU technique was applied to keep the solubility of the compounds stable. The ^1^H and ^13^C NMR spectra of the compounds were recorded with dynamic NMR method at 328.15 K by using the Topspin 2.1 NMR program. The internal standard was tetramethylsilane. IR spectra of the compounds were recorded with the FTIR-ATR method (400–4000 cm^–1^) on a IRSpirit Fourier transform (FT)-IR spectrophotometer (Shimadzu, Kyoto, Japan). Mass spectra of the samples were recorded using a liquid chromatography ion trap-time of flight tandem mass spectrometer (Shimadzu, Kyoto, Japan) equipped with an electrospray ionization (ESI) source, operating in both positive and negative ionization mode. For data analysis, Shimadzu’s LCMS Solution program was used. Electrothermal 9100 instrument (IA9100, Bibby Scientific Limited, Staffordshire, UK) was used to determine the melting points of the compounds. 

#### 2.1.1. Synthesis of compound 1 

**Figure 2:**
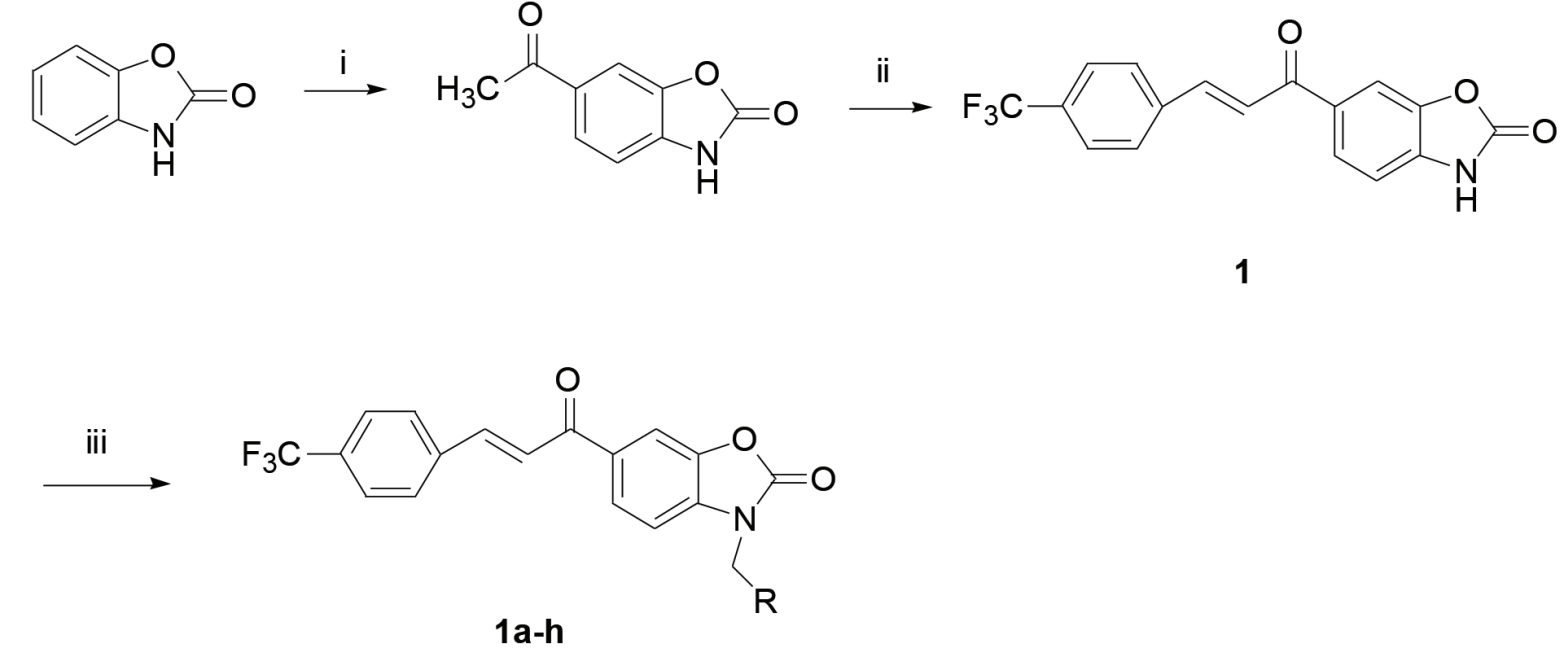
General synthetic pathway for compounds 1 and 1a – g. Reagent and conditions: (i) CH3COCl, AlCl3-DMF; (ii) 4-trifluoromethylbenzaldehyde, aq. KOH, EtOH; (iii) suitable amine, 37% formaldehyde, EtOH R: Dimethylamino (1a), Diethylamino (1b), Dipropylamino (1c), Pyrrolidino (1d), Piperidino (1e), Morpholino (1f), and N-methyl piperazino (1g).

The synthesis of the chalcone compound **1** , 6-[3-(4-trifluoromethylphenyl)-2-propenoyl]-2( *3H* )-benzoxazolone, was realized as described in detail in our previous study [27]. Briefly, 6-acetyl-2( *3H* )-benzoxazolone was synthesized by Friedel–Crafts reaction. Then, the chalcone compound **1** was synthesized by Claisen–Schmidt reaction that occurred between 6-acetyl-2( *3H* )-benzoxazolone and 4-trifluoromethyl benzaldehyde in basic condition as shown in Figure 2. 6-[3-(4-trifluoromethylphenyl)-2-propenoyl]-2( *3H* )-benzoxazolone was obtained with 80% yield. Physicochemical and spectroscopic characterizations of the compound **1** were reported in our previous study [27].

**(** E **)-6-{3-[4-(trifluoromethyl)phenyl]acryloyl}benzoxazol-2(** 3H **)-one (1)**

White powder, yield 80%. Mp: 257–259 °C. IR (cm^–1^) 1772, 1657, 1448, 1322, 1288, 1118, 1165, 1068, 815, 695. ^1^H NMR (400 MHz, DMSO-d_6_) δ (ppm) 8.12–8.14 (m, 3H, arom. H), 8.09 (d, 1H, Ar-CH=, *J* = 15.7 Hz), 8.06 (d, 1H, arom. H, *J* = 1.6 Hz), 7.80 (d, 2H, arom. H, *J* = 8.2 Hz), 7.78 (d, 1H, =CHCO, *J* = 15.7 Hz), 7.24 (d, 1H, arom. H, *J* = 8.2Hz). ^13^C NMR (100 MHz, DMSO-d_6_) δ (ppm) 187.7, 154.9, 143.9, 142.2, 139.2, 135.7, 131.9, 130.5, 129.9, 126.2, 124.9, 122.7, 119.1, 110.1. HRMS (ESI-MS) C_16_H_11_NO_3_ m/z calculated [M+H]^+^ 334.0686; measured 334.0687.

#### 2.2.2. Synthesis of Mannich bases, 1a–g

To the solution of 6-[3-(4-trifluoromethylphenyl)-2-propenoyl]-2( *3H* )-benzoxazolone (0.1 g, 0.3 mmol) in ethanol (4 mL), 37% formaldehyde solution (33 µL, 0.4 mmol) was added and heated. Then, the suitable seconder amine compound (0.1 mmol) was added to this mixture and refluxed for 20 min (Figure 2). After standing at room temperature, the mixture was crystallized, filtered, washed with ethanol, dried [50] and recrystallized from ethanol.

**3-[(Dimethylamino)methyl]-6-{3-[4-(trifluoromethyl)phenyl]acryloyl}benzoxazol-2(3H)-one (1a)**

Yellow powder, yield 65%. Mp: 179–181 °C. IR (cm^–1^) 1771, 1652, 1590, 1452, 1319, 1310, 1293, 1256, 1163, 1106, 1066, 926, 812, 694. ^1^H NMR (400 MHz, DMSO-d_6_) δ (ppm) 8.07 (4H, m, 4x Ar-CH), 8.02 (1H, d, *J* = 16.1 Hz, ol-CH), 7.76 (3H, m, 2xAr-CH, 1xol-CH), 7.44 (1H, bs, Ar-CH), 4.65 (2H, bs, N-CH_2_-N), 2.33 (6H, bs, 2x CH_3_). ^13^C NMR (100 MHz, DMSO-d_6_) δ (ppm) 187.76, 155.29, 144.31, 142.55, 136.89, 135.18, 132.75, 131.05, 129.39, 129.35, 128.71, 126.11, 122.23, 110.39, 110 02, 65.76, 42.70. HRMS (ESI-MS) C_20_H_17_F_3_N_2_O_3_ m/z predicted [M+H]^+ ^390.1191; found [M+H]^+^ 390.1181.

**3-[(Diethylamino)methyl]-6-{3-[4-(trifluoromethyl)phenyl]acryloyl}benzoxazol-2(3H)-one (1b)**

Yellow powder, yield 72%. Mp: 235–236 °C. IR (cm^–1^) 2977, 1762, 1668, 1618, 1324, 1269, 1121, 1068, 832, 608. ^1^H NMR (400 MHz, DMSO-d_6_) δ (ppm) 8.08 (2H, d, *J* = 8.0 Hz, Ar-CH), 8.04 (1H, d, *J* = 15.8 Hz, ol-CH), 7.92 (1H, s, Ar-CH), 7.78 (2H, d, *J* = 8.0 Hz, Ar-CH), 7.73 (1H, d, *J* = 15.8 Hz, ol-CH), 7.49 (1H, m, Ar-CH), 7.16 (1H, d, *J* = 6.89 Hz, Ar-CH), 4.75 (2H, s, N-CH_2_-N), 2.66 (4H, q, *J* = 6.9 Hz, N-CH_2_), 1.01 (6H, t, *J* = 7.0 Hz, 2x-CH_3_). ^13^C NMR (100 MHz, DMSO-d_6_) δ (ppm) 187.85, 160.67, 142.38, 139.46, 130.14, 126.51, 126.38, 126.35, 126.30, 126.11, 125.19, 123.40, 110.71, 110.18, 86.88, 62.37, 45.13, 12.92. HRMS (ESI-MS) C_22_H_21_F_3_N_2_O_3_ **** m/z predicted [M+H]^+ ^418.1504; found [M+H]^+^ 418.1490.

**3-[(Dipropylamino)methyl]-6-{3-[4-(trifluoromethyl)phenyl]acryloyl}benzoxazol-2(3H)-one (1c)**

Yellow powder, yield 37%. Mp: 157–158 °C. IR (cm^–1^) 2966, 2923, 2355, 1772, 1653, 1559, 1456, 1325, 1068, 1033, 831. ^1^H NMR (25 ºC, 400 MHz, DMSO-d_6_) δ (ppm) 8.10 (5H, m, 4x Ar-CH, 1x ol-CH), 7.78 (3H, m, 2x Ar-CH, 1x ol-CH), 7.41 (1H, d, *J* = 7.6 Hz, Ar-CH), 4.78 (2H, s, N-CH_2_-N), 2.61 (t, 4H, *J* = 6.50 Hz, N-CH_2_), 1.48 (m, 4H, 2x –CH_2_-), 0.84 (t, 6H, *J* = 7.20 Hz, 2x CH_3_). ^13^C NMR (25 ºC, 100 MHz, DMSO-d_6_) δ (ppm) 187.74, 155.26, 142.85, 142.04, 139.29, 136.82, 132.39, 129.79, 126.23, 126.06, 126.02, 125.87, 125.23, 110.27, 109.94, 63.46, 53.87, 20.64, 11.92. HRMS (ESI-MS) C_24_H_25_F_3_N_2_O_3_ m/z predicted [M+H]^+ ^446.1817; found [M+H]^+^ 446.1828.

**3-(Pyrrolidin-1-ylmethyl)-6-{3-[4-(trifluoromethyl)phenyl]acryloyl}benzoxazol-2(3H)-one (1d)**

Yellow powder, yield 85%. Mp: 179 °C. IR (cm^–1^) 2973, 2821, 1772, 1656, 1592, 1455, 1321, 1165, 1106, 1067, 814, 694.^ 1^H NMR (400 MHz, DMSO-d_6_) δ (ppm) 8.06 (5H, m, 4x Ar-CH, 1x ol-CH), 7.79 (2H, d, *J* = 8.9 Hz, 2xAr-CH), 7.73 (1H, s, Ar-CH), 7.54 (1H, bs, ol-CH), 4.84 (2H, s, N-CH_2_-N), 2.51 (4H, bd, *J* = 1.3 Hz, N-CH_2_-CH_2_), 1.69 (4H, s, CH_2_-CH_2_). ^13^C NMR (100 MHz, DMSO-d_6_) δ (ppm) 187.74, 153.35, 141.64, 139.39, 132.80, 129.73, 126.12, 126.07, 126.03, 126.01, 125.89, 125.45, 123,18, 110.15, 109.21, 65.21, 52.09, 22.31. HRMS (ESI-MS) C_22_H_19_F_3_N_2_O_3_ m/z predicted [M+H]^+ ^416.1348; found [M+H]^+^ 416.1349. 

**3-(Piperidin-1-ylmethyl)-6-(3-(4-(trifluoromethyl)phenyl)acryloyl)benzoxazol-2(3H)-one (1e)**

Yellow powder, yield 87%. Mp: 199–200 °C. IR (cm^–1^) 2936, 1771, 1659, 1608, 1447, 1319, 1288, 1110, 1067, 811, 697. ^1^H NMR (400 MHz, DMSO-d_6_) δ (ppm) 8.08 (5H, m, 4x Ar-CH, 1x ol-CH), 8.02 (1H, s, Ar-CH), 7.77 (2H, m, 2x Ar-CH), 7.48 (1H, bs, ol-CH), 4.71 (2H, s, N-CH_2_-N ), 2.62 (4H, s, N-CH_2_), 1.50 (4H, s, N-CH_2_-CH_2_), 1.34 (2H, s, -CH_2_). ^13^C NMR (100 MHz, DMSO-d_6_) δ (ppm) 187.82, 155.31, 142.05, 139.27, 137.11, 132.41, 129.78, 126.22, 126.07, 126.03, 126.98, 125.86, 125.24, 110.39, 109.76, 65.73, 51.55, 25.83, 23.89. HRMS (ESI-MS) C_23_H_21_F_3_N_2_O_3_ **** m/z predicted [M+H]^+ ^430.1504; found [M+H]^+^ 430.1495.

**3-(Morpholinomethyl)-6-{3-[4-(trifluoromethyl)phenyl]acryloyl}benzoxazol-2(3H)-one (1f)**

White powder, yield 89%. Mp: 215–217 °C. IR (cm^–1^) 2865, 2833, 1768, 1656, 1449, 1324, 1111, 1068, 814, 695. ^1^H NMR (400 MHz, DMSO-d_6_) δ (ppm) 8.15 (1H, d, *J* = 15.2 Hz, ol-CH), 8.12 (2H, d, *J* = 8.8 Hz, 2x Ar-CH), 8.10 (2H, d, *J* = 8.6 Hz, 2x Ar-CH), 8.08 (1H, s, 1x Ar-CH ), 7.79 (2H, m, Ar-CH, ol-CH), 7.55 (1H, d, *J* = 7.9 Hz, Ar-CH), 4.73 (2H, s, N-CH_2_-N), 3.20 (4H, m, 2x -CH_2_-O-), 2.66 (4H, m, 2x -CH_2_-N-). ^13^C NMR (100 MHz, DMSO-d_6_) δ (ppm) 187.87, 155.09, 142.61, 142.18, 139.29, 136.74, 132.67, 129.83, 126.26, 126.09, 126.06, 125.27, 123.18, 110.42, 110.11, 66.49, 64.96, 50.73. HRMS (ESI-MS) C_22_H_19_F_3_N_2_O_4_ **** m/z predicted [M+H]^+ ^432.1297; found [M+H]^+^ 432.1277.

**3-[(4-Methylpiperazin-1-yl)methyl]-6-{3-[4-(trifluoromethyl)phenyl]acryloyl} benzoxazol-2(3H)-one (1g)**

Yellow powder, yield 75%. Mp: 203–205 °C. IR (cm^–1^) 2940, 2800, 1772, 1658, 1592, 1449, 1322, 1288, 1163, 1110, 1068, 815, 695. ^1^H NMR (400 MHz, DMSO-d_6_) δ (ppm) 8.09 (5H, m, 4x Ar-CH, 1x ol-CH), 7.80 (3H, m, 2x Ar-CH, 1x ol-CH), 7.41 (1H, d, *J* = 7.6 Hz, Ar-CH), 4.73 (2H, s, N-CH_2_-N), 2.66 (4H, bs, 2x-N-CH_2_), 2.32 (4H, bs, 2x-CH_2_-N), 2.14 (3H bs, CH_3_). ^13^C NMR (100 MHz, DMSO-d_6_) δ (ppm) 187.82, 155.05, 142.60, 142.11, 139.28, 136.79, 132.55, 129.80, 126.23, 126.07, 126.03, 125.26, 123.17,110.40, 110.05, 64.81, 54.88, 50.21, 46.03. HRMS (ESI-MS) C_23_H_22_F3N_3_O_3_ m/z predicted [M+H]^+ ^445.1613; found [M+H]^+^ 445.1604.

### 2.2. Acetylcholinesterase inhibition assay

Acetylcholinesterase enzyme inhibition assay was performed according to our previous studies [4,41,51–55]. The inhibition effects of the compounds **1** and **1a–g** on AChE enzyme, obtained from electric eel [56], were recorded in accordance with Ellman’s method as demonstrated previously in detail [51,52,57]. The substrates of acetylthiocholine iodide (AChI) and 5,5’-dithiobis(2-nitro-benzoic acid) (DTNB) was used for cholinergic enzymatic reaction. For this purpose, 1 mL of Tris / HCl buffer (1.0 M, pH 8.0) and 10 μL of different concentrations of sample solution were dissolved in deionized water. Then, an aliquot of (50 μL) AChE enzyme was transferred and incubated at room temperature for 10 min. After the incubation period, an aliquot of DTNB (0.5 mM, 50 μL) was added. Then, the enzymatic reaction was started by adding 50 μL of AChI (10 mM). The breakdown of these substrates was monitored spectrophotometrically by the yellow color formation of 5-thio-2-nitrobenzoate anion as the result of the reaction of DTNB with thiocholine from hydrolysis of AChI with absorption at a wavelength of 412 nm [58,59].

### 2.3. Carbonic anhydrase inhibition assay

CA inhibition assay was recorded according to previous studies [4,10,27,49,60–64]. The hCA I and II isoenzymes were purified from human red blood cells by sepharose-4B-L-tyrosine-sulfanilamide affinity chromatography [65], which was used as an affinity matrix for selective retention of both hCA isoenzymes [27,66]. The inhibition effects of the compounds **1** and **1a–g** on both hCA isoenzymes were spectrophotometrically measured according to the previous method described in detail [49,60,67]. p-Nitrophenylacetate (PNA) was used as a substrate and changed to p-nitrophenolate ions (PNP). One CA isoenzyme unit is accepted as the amount of CA, which had absorbance change at 348 nm of PNA to PNP over a period of 3 min at 25 °C. After sepharose-4B-L-tyrosine-sulfanilamide affinity chromatography, the enzyme quantity was spectrophotometrically measured at 280 nm [68]. Moreover, the protein effluent was spectrophotometrically determined at 595 nm according to the Bradford method. Purity controls of both CA isoenzymes were performed according to the Laemmli procedure as described in previous studies. Both isoenzymes were visualized by two different acrylamide concentrations (10% and 3% acrylamide), containing 0.1% sodium dodecyl sulfate (SDS) [69,70].

### 2.4. Molecular docking studies

The most active compounds **1d** (pyrrolidine derivative) in terms of hCA I inhibition effect, **1g** ( *N* -methyl piperazine derivative) in terms of hCA II inhibition effect, and **1f** (morpholine derivative) in terms of AChE inhibition effect were docked at the binding sites of the mentioned enzymes to describe and confirm the inhibition effects of the newly synthesized compounds. The Protein Data Bank (PDB https://www.rcsb.org/) was used to get the structures of hCA I (4WR7), hCA II (3HS4), and AChE (1C2O) enzymes. The pdb files of the enzymes were prepared and transferred to AutoDockTools (ADT ver.1.5.6). Water molecules of the structures were removed and only polar hydrogen and Kollman charges were added to the proteins. Finally, the pdbqt files of the proteins were saved. 

The Drug Bank https://www.drugbank.ca/ was used to get the chemical structures of the reference drugs (AZA and TAC). First, the active sites of the AChE and hCA I/II enzymes were defined by using BIOVIA Discovery Studio Visualizer (v20.1.0.19295). Then, the reference drugs, acetazolamide (AZA) and tacrine (TAC), were docked into the human carbonic anhydrases (hCA I/hCA II) and AChE, respectively. Compounds **1d** , **1g** , and **1f** were drawn in ChemDraw (Professional, Version 19.0.1.28), passed to ChemDraw 3D (Professional, Version 19.0.1.28), and minimized. The molecules’ files were saved as pdb. Torsions of the compounds were examined and then compounds’ files ( **1d** , **1g** , and **1f)** were saved as pdbqt by AutoDockTools (ADT ver.1.5.6) [4].

AutoDockTools (ADT ver.1.5.6) was used for molecular docking studies. The Lamarckian genetic algorithm with local search (GALS) was used as a search engine, with a total of 10 runs. The active sites of enzymes were defined by a grid box of 70 × 70 × 70 points. Ten conformers of the compound were considered to evaluate the docking results. Finally, the conformer with the lowest binding free energy was evaluated using Python Molecule Viewer (PMV ver.1.5.6) and PyMOL (ver. 2.3.3, Schrodinger, LLC). 

### 2.5. Estimation of physicochemical and ADME properties

In silico prediction of the ADME parameters and physicochemical properties the compounds was performed using the SwissADME http://www.swissadme.ch/ web tool. The structures of the compounds **1** and **1a–g** were drawn and transformed to SMILES (simplified molecular-input line-entry system). Finally, the ADME parameters and physicochemical parameters of the compounds **1** and **1a–g** were calculated by running the program.

## 3. Results and discussion

### 3.1. Chemistry

The compounds **1** and **1a–g** were synthesized successfully for the first time (except **1** and **1g** ) [27,71] according to Figure 2. First, the chalcone compound **1** , 6-[3-(4-trifluoromethylphenyl)-2-propenoyl]-2( *3H* )-benzoxazolone, was synthesized according to our previous study [27] by the classical Claisen-Schmidt reaction realized between 4-trifluorobenzaldehyde and 6-acetyl-2( *3H* )-benzoxazolone [10,27]. In the second step, the Mannich bases were synthesized by the Mannnich reaction of compound **1** with suitable seconder amines. The amine compounds used were as follows: dimethylamine ( **1a** ), diethylamine ( **1b** ), dipropylamine ( **1c** ), pyrrolidine ( **1d** ), piperidine ( **1e** ), morpholine ( **1f** ), and 1-methyl piperazine ( **1g** ). The compound synthesized with the highest yield was morpholine derivative compound **1f** (89%) while the compound synthesized with the lowest yield was dipropylamine derivative compound **1c** (37%) in the series.

According to IR spectra of the compounds, the lactam and ketone C=O stretching bands were seen about 1770 cm^–1^ and 1650 cm^–1^. According to ^1^H NMR spectra of the synthesized compounds **,** the methylene protons between two nitrogen atoms of Mannich bases appeared at the area of 4.5–5.5 ppm as expected. On the other hand, ^1^H NMR spectra of the compounds showed that all compounds were configured *trans* , as understood from coupling constant *J* = 15.2 – 16.1 Hz for vinyl protons or from the results of the NOESY NMR of the compounds. The DEPT NMR spectra of the compound **1a** were recorded to determine primary, secondary, and tertiary carbon atoms of the series. According to the DEPT NMR results of the compound **1a** , carbons of the carbonyl groups were seen about 187 ppm (ketone) and 155 ppm (lactam). Signals of the other quaternary carbons were seen at 142.55, 136.89, 135.18, 132.75, 128.71, and 110.02 ppm. In the aliphatic region, the carbon of methylene group (secondary) was seen as a negative signal while the carbons of methyl groups (primary) were seen as a positive signal. The chemical structures of the newly synthesized compounds **1, 1a–g** were confirmed and characterized by IR, ^1^H NMR, ^13^C NMR, and HRMS (see the experimental part for details).

### 3.2. Carbonic anhydrases inhibitory activities

The CA inhibition effects of the compounds **1a–g** were reported for the first time in this study, and the CA inhibition results are shown in Table 1. Acetazolamide (AZA) was used as a reference drug, and its K_i_ values were 84.4 ± 8.4 nM towards hCA I and 59.2 ± 4.8 nM towards hCA II.

**Table 1:** Inhibition effects of the compounds 1 and 1a–g on hCA I, hCAII, and AChE enzymes.

Compounds	Ki (nM)		
	hCA I*	hCA II*	AChE*
1	154.0 ± 9.3	33.6 ± 4.5	158.9 ± 33.5
1a	31.4 ± 5.3	23.3 ± 2.0	129.9 ± 17.6
1b	36.8 ± 7.7	37.6 ± 4.3	142.1 ± 22.1
1c	22.3 ± 2.3	24.6 ± 4.7	115.8 ± 32.0
1d	12.3 ± 1.2	19.0 ± 3.0	97.6 ± 14.4
1e	27.6 ± 2.9	18.1 ± 3.5	84.0 ± 19.2
1f	20.4 ± 1.7	41.0 ± 5.5	35.2 ± 2.0
1g	53.0 ± 1.8	8.6 ± 1.9	48.5 ± 10.2
AZA**	84.4 ± 8.4	59.2 ± 4.8	-
TAC***	-	-	68.6 ± 3.8

* Mean from three different assays.

The compounds **1** and **1a–g** had K_i_ values of in the range of 12.3 ± 1.2 to 154.0 ± 9.3 nM towards hCA I (Table 1). According to hCA I inhibitory activity results of the compounds, all Mannich bases synthesized ( **1a–g)** had higher inhibitory activities than the reference compound, AZA. On the other hand, compound **1** had both the lowest inhibitory activity in the series and lower inhibitory activity than the reference compound, AZA. As a result, the aminomethylation from the 3rd position of the benzoxazolone ring by Mannich reaction caused a significant increase (in the range of 2.1–12.5 times) in hCA I inhibition effects, as expected. This result suggested that the prevention of tautomerization between the nitrogen atom and carbonyl of the benzoxazolone ring had a crucial role in hCA I inhibitory activity. According to the K_i_ values presented in Table 1, the pyrrolidine derivative compound **1d** had the highest inhibition effect in the series with a lower K_i_ value (12.3 ± 1.2 nM) than AZA (K_i_ = 84.4 ± 8.4 nM) towards hCA I. Including the nitrogen atom of Mannich base in a cyclic structure (pyrrolidine derivative compound **1d** ) led to an approximately three-fold increase in hCA I inhibitory activity comparing to diethylamine derivative compound **1b** . On the other hand, replacing the pyrrolidine ring (compound **1d** ) with a six-membered ring (piperidine derivative compound **1e** ) led to an approximately two-fold decrease in hCA I inhibitory activity. Comparing the K_i_ values of compounds **1e** and **1f** pointed out that the replacement of the carbon atom on the piperidine heterocycle with the oxygen atom caused an increase in the hCA I inhibition effect. This may be due to the formation of a hydrogen bond between the oxygen atom and the active site of hCA I isoenzyme. 

According to Table 1, K_i_ values of the compounds **1, 1a–g** were in the range of 8.6 ± 1.9 to 41.0 ± 5.5 nM towards hCA II isoenzyme. The hCA II inhibitory activity results of the compounds presented in Table 1 showed that all compounds synthesized ( **1** and **1a–g** ) showed higher inhibition effects with their lower K_i_ values than the reference compound AZA. Thus, the results presented in Table 1 pointed out that the aminomethylation from the 3rd position of the benzoxazolone ring by Mannich reaction generally resulted in an increase (except compounds **1b** and **1f** ) in hCA II inhibitory activity. The most potent compound in the series was *N* -methyl piperazine derivative compound **1g** with the remarkable K_i_ value of 8.6 ± 1.9 nM, which is approximately 7 times lower than AZA. Thus, compound **1g** was the most important compound of the series in terms of hCA II inhibitory activity and can be regarded as a lead molecule for further investigations. According to Table 1, the morpholine derivative compound **1f** had the lowest hCA II inhibition effect in the series with the K_i_ value of 41.0 ± 5.5 nM which is 1.4 times lower than AZA. The pyrrolidine derivative compound **1d** including the nitrogen atom of Mannich base in a cyclic structure had higher inhibitory activity (K_i _= 19.01 ± 3.0 nM) about 2 times towards hCA I than diethylamine derivative compound **1b** (K_i_ = 37.6 ± 4.3 nM). Comparing the K_i_ values of compounds **1e** and **1f** towards hCA II showed that the replacement of the carbon atom on the piperidine heterocycle with the oxygen atom led to a decrease about 2 times in the hCA II inhibition effect. On the other hand, the inhibition results in Table 1 suggested that the compounds carrying the nitrogen atom of Mannich base in a cyclic structure (compounds **1d - g** ) generally had higher inhibitory activities towards hCA II isoenzyme than the compounds carrying the nitrogen atom of Mannich bases on a straight chain (compounds **1a–c** ). 

### 3.3. AChE inhibitory activities

The AChE inhibitory activities of the newly synthesized compounds **1** and **1a–g** were reported for the first time in this study and the results were presented in Table 1. The reference drug used was tacrine (TAC) and its K_i_ value was 68.6 ± 3.8 nM towards AChE enzyme. According to the results presented in Table 1, K_i_ values of the compounds **1** and **1a–g** were in the range of 35.2 ± 2.0 to 158.9 ± 33.5 nM towards AChE. The inhibition results showed that compounds **1f** and **1g** had higher inhibitory effects than the reference drug TAC. The morpholine derivative compound **1f** was the most active compound in the series with the K_i_ value as 35.2 ± 2.0 nM. On the other hand, the chalcone compound **1** had the lowest inhibition effect in the series with the K_i_ value as 158.9 ± 33.5 nM. Thus, the inhibition results showed that synthesis of Mannich derivatives from the 3rd position of the benzoxazole ring of the compound **1** was a modification that contributed to the AChE inhibition effect. This may be due to the prevention of tautomerization between the amino and carbonyl groups of the benzoxazolone ring. Besides, the inhibition results presented in Table 1 pointed out that the compounds where the nitrogen atom of Mannich base was included in a cyclic structure (compounds **1d - g** ) had higher inhibitory effects on AChE enzyme than the compounds carrying the nitrogen atom of Mannich bases on a straight chain (compounds **1a–c** ). Even, adding one more heteroatom (oxygen or nitrogen) to this heterocyclic structure (compounds **1f** and **1g** , respectively) resulted in an increase in AChE inhibitory activity comparing to piperidine derivative compound **1e** . 

### 3.4. Molecular docking studies

To demonstrate the binding model of the synthesized compounds **1** and **1a–g** with the active sites of hCA I, hCA II, and AChE enzymes, docking studies were performed using AutoDockTools (ADT ver.1.5.6). The X-ray crystallographic structures of hCA I (4WR7), hCA II (3HS4), and AChE (1C2O) were obtained from the Protein Data Bank https://www.rcsb.org/. The chalcone compound **1** and the most potent compounds **1d** (pyrrolidine derivative), **1g** ( *N* -methyl piperazine derivative), and **1f** (morpholine derivative) in terms of hCA I, hCA II, and AChE inhibitory activities (respectively) were docked at the binding sites of the mentioned enzymes to describe and confirm the inhibition effects of the series.

First, the active sites of the hCA I, hCA II, and AChE were defined by using BIOVIA Discovery Studio Visualizer. Then, the reference drugs acetazolamide (AZA) and tacrine (TAC) were docked into the human carbonic anhydrases (hCA I and hCA II) and AChE, respectively. These simulations were done successfully and the binding free energy scores of the reference drugs were found as –6.53 (in 4WR7), –6.77 (in 3HS4), and –7.10 (in 1C2O) kcal / mol. Finally, the binding free energy scores and K_i_ values of the chalcone compound **1** and the most potent compounds **1d** , **1g** , and **1f** in the series were calculated by docking simulations and the results were presented in Table 2. 

**Table 2:** Docking results of the compounds 1, 1d, 1g, and 1f.

	hCA I		hCA II		AChE	
Compound	Energy scrore	Ki (µM)	Energy scrore	Ki (µM)	Energy scrore	Ki (nM)
1	–6.62	14.01	–7.43	3.6	–8.82	342.12
1d	–8.18	1.01	-	-		
1g	-	-	–8.05	1.25	-	-
1f	-	-			–10.17	35.38
AZA*	–6.53	16.39	–6.77	10.97		
TAC**	-	-	-	-	–7.1	6.3 µM

*Acetazolamide (AZA) was used as a reference compound for both hCA I (4WR7) and hCA II (3HS4).**Tacrine (TAC) was used as a reference compound for AChE (1C2O).

**Figure 3:**
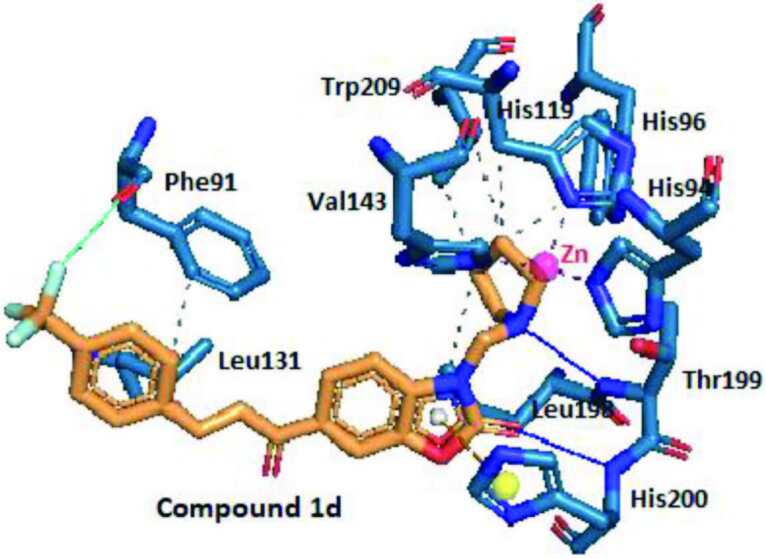
Three-dimensional representation of compound 1d, showing its interaction with the active site of hCA I. Grey color represents hydrophobic interactions, blue color represents hydrogen bondings, green color represents halogen bonds, and red represents π-cation interactions.

The energy score of the most active compound **1d** (pyrrolidine derivative) towards hCA I was found as –8.18 kcal/mol. Thus, this suggested the strong interactions of the compound with the active site of the 4WR7, as expected. According to Figure 3, the carbonyl moiety of the benzoxazolone ring of the compound **1d** realized a hydrogen bonding with the amino groups of the amino acids His200. This showed the importance of the prevention of tautomerization between the nitrogen atom and carbonyl of the benzoxazolone ring by Mannich reaction in designing hCA I inhibitory compounds in terms of the strong interactions with the enzyme. Besides, the pyrrolidine ring realized a hydrophobic interaction with the amino acid Thr199. According to the binding pattern of compound **1d** presented in Figure 3, the π-cation interaction was exhibited between the benzoxazolone ring of the compound and the side chain of the amino acid His200. On the other hand, seven hydrophobic interactions were exhibited between the compound **1d** and the amino acids Phe91, His119, Leu131, Val143, Leu198, and Trp209. Furthermore, trifluoromethyl moiety of the compound **1d** involved in a halogen bond with the amino acid Phe91. This pointed out that the trifluoromethyl group of the compounds designed had an important role in strong interactions with the active site of the hCA I isoenzyme, as planned.

**Figure 4:**
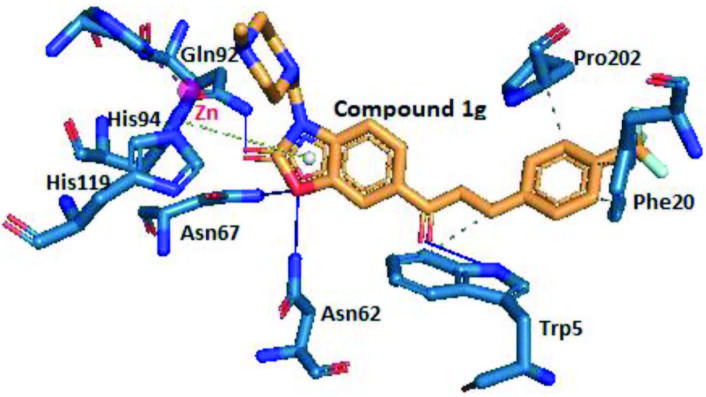
Three-dimensional representation of compound 1g, showing its interaction with the active site of hCA II. Grey color represents hydrophobic interactions, blue color represents hydrogen bondings, and green represents π-stacking interactions.

Compound **1g** , *N* -methyl piperazine derivative, showed higher inhibitory activity (K_i_ = 8.6 ± 1.9 nM) than AZA towards hCA II and its docking score was –8.05 kcal/mol that is lower than the reference drug’s binding free energy (–6.77 kcal/mol), as expected. The binding pattern of compound **1g** with the active site of the hCA II enzyme (3HS4) is shown in Figure 4. In Figure 4, it was seen that the carbonyl group of benzoxazolone heterocycle of compound **1g** realized a hydrogen bond with the side chain of the amino acid Gln92. This shows how the prevention of tautomerization between amino and carbonyl groups by aminomethylation contributes positively to hCA II inhibitory activity. Furthermore, two hydrogen bonds were observed between the oxygen atom of the benzoxazolone ring and the amino acids Asn62 and Asn67. On the other hand, the carbonyl group of chalcone structure of compound **1g** realized a hydrogen bond with the side chain of the amino acid Trp5. According to the binding profile of compound **1g** (Figure 4), π-stacking interaction was exhibited between the benzoxazolone ring of the compound and the amino acid His94. Besides, four hydrophobic interactions were seen between the compound **1g** and the amino acids Trp5, Phe20, and Pro202. 

**Figure 5:**
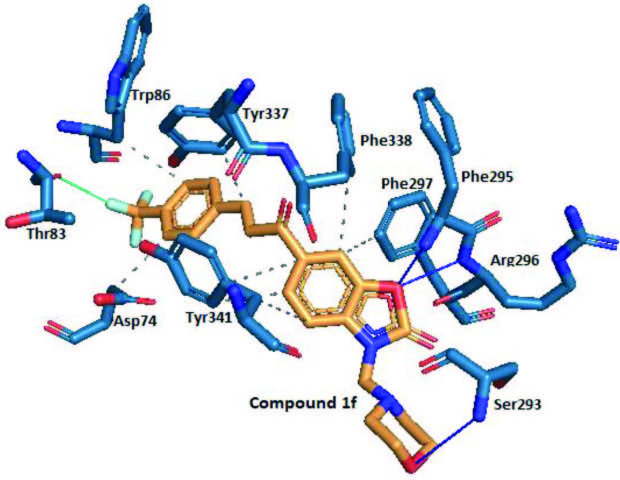
Three-dimensional representation of compound 1f, showing its interaction with the active site of AChE. Grey color represents hydrophobic interactions, blue color represents hydrogen bondings, and the green represents halogen bonds.

The morpholine derivative compound **1f** had the highest inhibition effect on AChE enzyme in the series and docked in 1C2O to explain its inhibition effect. As expected, compound **1f** had lower energy score, –10.17 kcal/mol, in AChE than reference drug TAC (–7.10 kcal/mol), recommending a strong interaction between the compound **1f** and the 1C2O. According to Figure 5, the oxygen atom on the first position of the benzoxazolone ring interacted with the enzyme via two hydrogen bonds with the amino groups of the amino acids Phe295 and Arg296. Similarly, the oxygen atom of the morpholine moiety formed a hydrogen bond with the amino group of Ser293. Thus, this suggested that aminomethylation of benzoxazolone ring with morpholine was a useful modification in terms of strong interactions with the active site of AChE. On the other hand, eight hydrophobic interactions were detected between compound **1f** and the amino acids Asp74, Trp86, Phe297, Tyr337, Phe338, and Tyr341 (Figure 5). Additionally, one halogen bond was exhibited between the trifluoromethyl group of the compound **1f** and the side chain of the amino acid Thr83. This pointed out the importance of the trifluoromethyl moiety in designing in terms of AChE inhibition effect. 

### 3.5. Estimation of physicochemical properties

Both the physicochemical and the ADME properties of a compound are very crucial in oral drug-candidate designing. ‘Drug-likeness’ is a term that describes the potentiality for a compound to be an oral drug in terms of bioavailability [72]. Lipinski’s 5 rules are used to evaluate this potentiality of a molecule [4,73]. In this study, both the physicochemical and the pharmacokinetic properties of the synthesized compounds **1** and **1a–g** were estimated using the SwissADME http://www.swissadme.ch/index.php web tool [4,72]. For this purpose, molecular weights, the numbers of hydrogen bond acceptors and donors, the topological surface areas (TPSA; a sum of polar atoms’ surfaces), lipophilicities, water solubilities, and bioavailability of the synthesized compounds **1** and **1a–g** are presented in Table 3.

**Table 3:** In silico physicochemical and pharmacokinetic properties of the compounds 1 and 1a – g.

Compound	MWa	HBAb	HBDc	TPSAd	CLogPo/we	logSf	Bioavailability scoreg
1	333.26	6	1	63.07	3.88	- 4.49	0.55
1a	390.36	7	0	55.45	3.97	- 4.85	0.55
1b	418.41	7	0	55.45	4.57	- 5.33	0.55
1c	446.46	7	0	55.45	5.29	- 6.01	0.55
1d	416.39	7	0	55.45	4.28	- 5.29	0.55
1e	430.42	7	0	55.45	4.57	- 5.59	0.55
1f	432.39	8	0	64.68	3.70	- 4.83	0.55
1g	445.43	8	0	58.69	3.75	- 5.02	0.55

a Molecular weight (<500 Da), b Number of hydrogen bond acceptors (<10), c Number of hydrogen bond donors (<5), d Topological polar surface area (20–130 Å2), e Octanol/water partition coefficient (Consensus logP value, recommended range: −2.0 to 6.5), f Aqueous solubility prediction (not higher than 6), g Abbott bioavailability score (probability at least 10% oral bioavailability in rat).

The results in Table 3 showed that all of the synthesized compounds were in agreement with Lipinski’s rule of 5. The compounds’ ClogP values ranged between 3.70 and 5.29 (˂6.5), molecular weight range of 333.26–446.26 (˂500), HBA range of 6–8 (≤ 10), and HBD values 0–1 (˂5), suggesting that compounds **1** and **1a–g** have desired drug-likeness properties. On the other hand, all of the compounds had desired logP values and were estimated to have high gastrointestinal absorption and to penetrate the blood-brain barrier. These are advantages for the oral use of the synthesized compounds as AChE inhibitors in AD.

**Figure 6:**
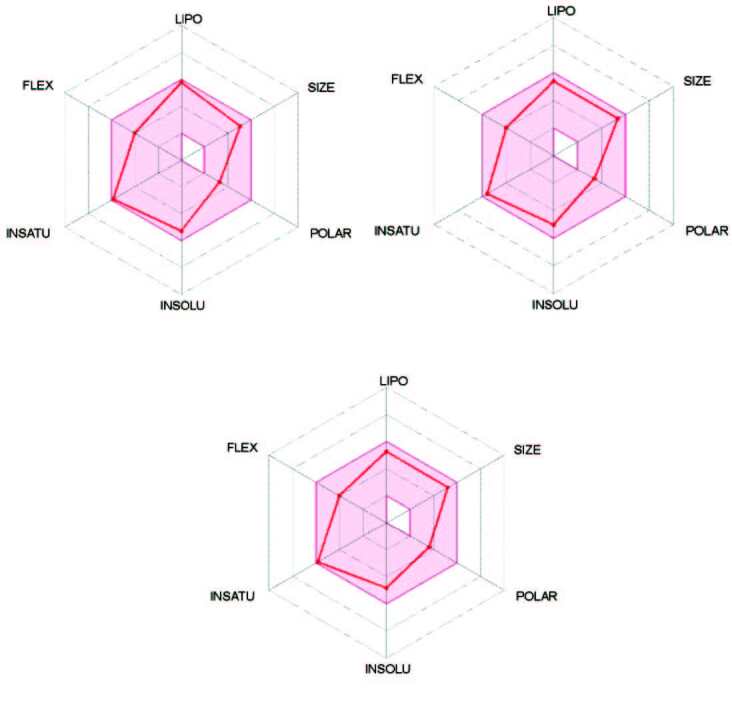
Bioavailability radar charts of compounds 1d, 1g, and 1f.

The radar charts of the most potent compounds **1d, 1g,** and **1f** towards hCA I, hCA II, and AChE, respectively, are shown in Figure 6. The pink-colored areas in these charts demonstrate the ideal ranges for each physicochemical properties; LIPO (lipophilicity, XLogP: between –0.7 and +5.0), size (MW: between 150 and 500 g/mol), POLAR (polarity, TPSA: between 20 and 130 Å^2^), INSOLU (solubility, log S: not higher than 6), INSATU (saturation; the fraction of carbons in the sp^3^ hybridization not less than 0.25), FLEX (flexibility, no more than 9 rotatable bonds) [72]. The radar charts presented in Figure 6 pointed out that the physicochemical properties of the most potent compounds **1d** , **1g** , and **1f** were fully located in the pink-colored area. As a result, the leading compounds **1d** , **1g** , and **1f** of the series were predicted as oral bioavailable drug candidates due to having promising pharmacokinetic properties.

## 4. Conclusion

The designed compounds **1a–g** , 3-(aminomethyl)-6-{3-[4-(trifluoromethyl)phenyl]acryloyl}-2( *3H* )-benzoxazolones, were synthesized and purified successfully. Their inhibition effects on hCA I, hCA II, and AChE enzymes were investigated for the first time. The inhibition results of the newly synthesized compounds showed that all Mannich bases had higher inhibitory activities against carbonic anhydrases (hCA I and hCA II) than the reference compound AZA. The pyrrolidine derivative compound **1d** had the highest inhibition effect in the series on hCA I with a lower K_i_ value (12.3 ± 1.2 nM) than AZA (K_i_ = 84.4 ± 8.4 nM). Besides, the most potent compound in the series was the *N* -methyl piperazine derivative compound **1g** having K_i_ value as 8.55 ± 1.90 nM towards hCA II. According to the hCA inhibition results, compounds **1d** and **1g** were the most expressive lead compounds of the study with remarkable K_i_ values (12.3 nM and 8.55 nM, respectively) which are approximately 7 times lower than AZA. According to the AChE inhibitory activity results, the morpholine derivative compound **1f** was the most active compound in the series with the K_i_ value as 35.2 ± 2.0 nM, which is about two-fold lower than the reference drug, TAC. On the other hand, the most active compounds **1d** (pyrrolidine derivative) towards hCA I, **1g** ( *N* -methyl piperazine derivative) towards hCA II, and **1f** (morpholine derivative) towards AChE were docked at the binding sites of the mentioned enzymes to explain the inhibitory activities of the compounds. The docking results showed that the compounds **1d** , **1g** , and **1f** had strong interactions with the active sites of hCA I, hCA II, and AChE, as expected. ADME prediction studies of the compounds **1** and **1a–g** pointed out that the newly synthesized Mannich bases were not only potent AChE and CAs inhibitory compounds but also had promising physicochemical and ADME properties for further investigations. As a conclusion, in vitro inhibition results and in silico studies of the synthesized compounds showed that the aminomethylation from the 3rd position of the benzoxazolone ring of the chalcone compound **1** by Mannich reaction is a useful modification in terms of carbonic anhydrases and AChE inhibitory activities as well as optimization of physicochemical and pharmacokinetic properties.
